# Packaging and Uncoating of CRISPR/Cas Ribonucleoproteins for Efficient Gene Editing with Viral and Non-Viral Extracellular Nanoparticles

**DOI:** 10.3390/v15030690

**Published:** 2023-03-06

**Authors:** Dmitriy Mazurov, Lama Ramadan, Natalia Kruglova

**Affiliations:** 1 Cell and Gene Technology Group, Center for Precision Genome Editing and Genetic Technologies for Biomedicine, Institute of Gene Biology RAS, 119334 Moscow, Russia; 2 Phystech School of Biological and Medical Physics, Moscow Institute of Physics and Technology, 141701 Moscow, Russia

**Keywords:** CRISPR, Cas9 ribonucleoprotein, virus-like particles, exosomes, HIV assembly and disassembly, delivery

## Abstract

Rapid progress in gene editing based on clustered regularly interspaced short palindromic repeats/CRISPR-associated protein (CRISPR/Cas) has revolutionized functional genomic studies and genetic disease correction. While numerous gene editing applications have been easily adapted by experimental science, the clinical utility of CRISPR/Cas remains very limited due to difficulty in delivery to primary cells and possible off-target effects. The use of CRISPR in the form of a ribonucleoprotein (RNP) complex substantially reduces the time of DNA exposure to the effector nuclease and minimizes its off-target activity. The traditional electroporation and lipofection methods lack the cell-type specificity of RNP delivery, can be toxic for cells, and are less efficient when compared to nanoparticle transporters. This review focuses on CRISPR/Cas RNP packaging and delivery using retro/lentiviral particles and exosomes. First, we briefly describe the natural stages of viral and exosomal particle formation, release and entry into the target cells. This helps us understand the mechanisms of CRISPR/Cas RNP packaging and uncoating utilized by the current delivery systems, which we discuss afterward. Much attention is given to the exosomes released during viral particle production that can be passively loaded with RNPs as well as the mechanisms necessary for particle fusion, RNP release, and transportation inside the target cells. Collectively, together with specific packaging mechanisms, all these factors can substantially influence the editing efficiency of the system. Finally, we discuss ways to improve CRISPR/Cas RNP delivery using extracellular nanoparticles.

## 1. Introduction

Discovered in the K12 strain of *Escherichia coli* bacteria over 30 years ago [1], CRISPR sequences initially provided researchers with information about the phylogenetic diversity of bacterial strains [2]. Much later, CRISPR was recognized as an adaptive antiviral immune system in bacteria, archaea (reviewed in [3]), and even bacteriophages [4]. Its adaptation for DNA editing in human cells by using CRISPR-associated protein 9 (Cas9) and single-guide RNA (sgRNA) [5,6,7] has revolutionized genome studies and genetic disease treatment. However, the difficulties of CRISPR/Cas delivery into clinically relevant primary human cells and their inherent resistance to homology-directed repair (HDR)-based DNA modification [8] significantly impede therapeutic applications of CRISPR/Cas.

Cas9 and sgRNA can be delivered to cells or tissues in the form of DNA, RNA, or RNP. The delivery methods can be divided into two groups: viral (lentiviral, adenoviral, and adeno-associated viral vectors) and non-viral, which can be further subdivided into chemical (lipid encapsulation, polymeric nanoparticles, gold nanoparticles, and modification with cationic or cell-penetrating peptides (CPP)), physical (electroporation and microinjection), and extracellular vesicle-based methods [9]. Chemical and physical techniques provide a sufficient level of transfection but are invasive and restricted to ex vivo applications, although some chemical delivery systems have recently been used to treat cancers in vivo [10]. Delivering CRISPR/Cas by transfecting cells with plasmid DNA has several clear disadvantages. First, the external dsDNA can potentially be integrated into the host chromosome. Second, the prolonged expression of Cas9 nuclease results in the accumulation of many off-target effects. Finally, plasmid DNA is toxic, especially for primary cells [3,11]. RNA transfection reduces the level and time of Cas9 protein expression. It can be delivered by the same methods used for DNA transfection. However, both DNA and RNA are capable of inducing cellular innate responses leading to the premature death of the primary cells [12]. RNPs lack these indicated shortcomings and thus became very popular in editing primary cells. However, the large size and weak overall charge of Cas RNP reduce the efficacy of the complex transfer to the cytoplasm and especially the nucleus using traditional electroporation or cationic lipids [13].

The packaging of CRISPR/Cas RNP into nanoparticles of viral or cellular origin can overcome these limitations and advance its delivery both ex vivo and in vivo. In contrast to electroporation, which results in a high degree of cell death, viral methods of RNP delivery are much gentler, their efficacy is easily adjustable via particle concentration/titration, and the target specificity can be changed using different envelopes (Envs) [14]. Despite the anticipated specificity and efficiency of complex recruitment, the existing lentiviral, retroviral, and exosomal Cas9 RNP delivery systems are far from optimal. To better understand the degree of particle assembly/disassembly interrogation due to the packaging of large Cas9 RNPs, we first reviewed the literature on natural mechanisms of retroviral and exosomal particle biogenesis and small reporter protein delivery. Then, we critically analyzed the specific and non-specific mechanisms of Cas9 RNPs recruitment and release utilized by different nanoparticle systems, and raised an important question about extracellular vesicles co-purified with viral particles that may affect the editing capacity of some systems. Finally, we outlined the parameters and rules that are important for the best particle-based Cas RNP designs and perspective approaches that would help improve the existing systems.

## 2. Retroviral Assembly with a Cargo Protein: What Was Known Prior to the CRISPR/Cas Era

Prior to CRISPR editing tool development, large quantities of data on protein cargo delivery using pseudoviruses had been accumulated that further facilitated the engineering of viral particles loaded with Cas9 RNP. However, to better understand the mechanisms of artificial system assembly, we first briefly describe the natural mechanisms of retroviral particle formation.

### 2.1. Lentiviral Particle Assembly

All retroviruses, including the human immunodeficiency virus (HIV), have similar mechanisms of viral particle assembly. The structural core protein of retroviruses, called Gag, has an intrinsic propensity for self-multimerization and the formation of spherical nanoparticles. It does not require the presence of any other viral or cellular proteins in vitro; however, it needs phospholipids for particle-like structure assembly [15] as well as its interaction with RNA, which greatly enhances this process [16]. In the absence of viral packaging RNA, “empty” particles produced from cells non-specifically incorporate cellular RNAs [16,17]. The N-terminal portion of Gag contains a basic amino acid stretch that serves as a signal for glycine myristylation at the second position of the polyprotein. Mutating G2 to any other amino acid abolishes particle formation [18]. It should be noted that although Gag is a self-assembling protein, replication-competent retroviruses incorporate two copies of genomic RNA and non-structural enzymatic viral proteins. The absence of these viral components in particles may substantially reduce the titer of the produced virions and their infectivity. Gag consists of four proteins: matrix (p17, MA), which is responsible for anchoring Gag to the membrane and Env recruitment; capsid (p24, CA), which mediates Gag multimerization; nucleocapsid (p7, NC), which is required for viral RNA incorporation and Gag assembly; and p6, which participates in viral protein R (Vpr) binding and virion fission (reviewed by Eric Freed in [19]). Following budding, HIV viral protease cleaves Gag into p17, p24, p7, and p6 proteins and the spacer peptides SP1 and SP2, triggering core condensation into a conically shaped structure, a process called virion maturation. The level of protease that is expressed from the *gag-pol* gene as a result of ribosome frameshifting is 20 times lower than the amount of Gag product and is important for proper virus maturation. The overexpression of HIV protease can drastically reduce virus infectivity [20,21] and even induce the off-target cleavage of a cargo protein, which is discussed below. However, the fusion of HIV protease with Vpr and/or use of a less active protease mutant substantially reduces the protease activity that can be applied for the generation of a “super-split” safe lentiviral system [22]. In addition, the proteolytic cleavage of Gag modulates the interaction between the cytoplasmic domain of the viral Env and the matrix protein, increasing the fusogenic activity of the HIV Env [23,24]. Finally, the maturation process allows viruses to complete both the entry and uncoating steps of replication. For safety reasons, some engineered virus-like particles (VLPs) contain immature Gag. Therefore, it should be remembered that such VLPs may have reduced entry capacity and targeted cargo delivery when pseudotyped with their own Env.

### 2.2. Packaging Reporter Proteins with Gag

C-type retroviruses such as HIV and murine leukemia virus (MLV) are the most popular viruses for molecular engineering as they bud from the plasma membrane of producer cells. This facilitates the pseudotyping of viral particles with different Envs of interest and the incorporation of cargo proteins translated in the cytosol. Gag assembly is very sensitive to modifications and protein insertions. From the Gag protein structure described above, it becomes clear that the addition of a reporter or cargo protein to the N-terminus of Gag will abolish its myristoylation and assembly. The attachment of these proteins to the C-terminal of Gag is less harmful to its assembly and is widely used to study Gag trafficking alone [25,26], although the formed particles have been reported to have an irregular structure [27]. Nevertheless, the close proximity to the p6 region responsible for binding Gag to the endosomal sorting complexes required for transport (ESCRT) proteins, particle release, as well as the overlap with the *pol* gene, exclude the use of Gag fused to the green fluorescent protein (GFP) in the context of the full viral genome. The embedding of fluorescent reporter proteins within different domains of Gag (for example, at the end of the MA) is also a difficult task, often resulting in the generation of replication-defective virions [28]. One of the first examples of a successfully engineered fluorescent replication-competent retrovirus was HIV-1 iGFP, where GFP was placed in between the MA and CA domains of Gag and flanked by viral protease cleavage sites [29]. It maintained a multiplicity of replication, although at a reduced capacity compared to an unmodified virus. This molecular clone served as an excellent tool for the live imaging of replicating HIV-1 and understanding the mechanisms of cell-to-cell transmission in both conventional cell systems [30] and 3D tissue cultures [31]. Adding a protein of interest to the sequence of Gag generally reduces the titer of produced VLPs, the degree of which varies depending on the Gag-Pol intervention site and the type and size of the loaded protein [32]. To restore viral particle production, researchers co-transfect plasmids encoding modified Gag with those containing intact Gag, a process known as viral complementation. By adjusting the ratio of these two plasmids, it is possible to achieve an optimal balance between the VLP payload and titer. Thus, if the number of HIV Gag molecules per particle is 2000 on average, then the co-transfection of Gag-YFP (yellow fluorescent protein) with the Gag expression plasmid at a 1:1 molar ratio will yield VLPs with a 50% YFP payload level [33]. Attaching the reporter protein to the N-terminus of Gag is still possible. Jun Komano’s lab published a series of studies in which the MA myristylation signal was successfully substituted with that from Lyn kinase (LM) or the phospholipase C-δ1 pleckstrin homology (PH) domain, which was then attached to the N-terminus of the reporter protein GFP or β-lactamase (BlaM) and placed upstream of Gag-Pol [34,35,36]. This design has a clear advantage over C-terminal Gag modification because it maintains natural VLP maturation and efficient reporter protein transduction. However, changes in the HIV matrix protein sequence/structure as mentioned can reduce HIV Env incorporation and its fusogenic potential, which will complicate ex vivo or in vivo protein delivery to specific targets such as CD4-lymphocytes.

### 2.3. Vpr-Based Packaging of Reporter Proteins

A conceptually different method of reporter protein encapsidation involves the fusion of cargo with a small HIV accessory protein, Vpr, which specifically binds to the p6 domain of Gag [37,38]. The incorporation is efficient when GFP is attached to the N-terminus [39] or C-terminus of full-length Vpr [40], or to the N-terminal (1–71 amino acids) portion of Vpr [41]. BlaM-Vpr was one of the first efficient and popular systems distinguishing viral particle attachment from their fusion. It consists of the enzyme β-lactamase fused to the N-terminus of Vpr. Following the attachment and fusion of VLPs with CCF2/AM preloaded target cells, BlaM is released into the cytoplasm and cleaves the β-lactam ring of the CCF2, changing its emission spectrum from green to blue [42]. A certain advantage of packaging proteins using Vpr is its minimal influence on the Gag assembly process and the elimination of the need to complement viral particle production with helper vectors. However, normally, only ~275 molecules of Vpr are incorporated into a single native HIV-1 virion [43]; in other words, the Vpr to Gag ratio is approximately 1:7 [44], which may provide an insufficient payload for certain systems. The expression of Vpr from a separate plasmid and an increase in the ratio of Vpr to a packaging plasmid can improve the payload to some extent.

**Figure 1:**
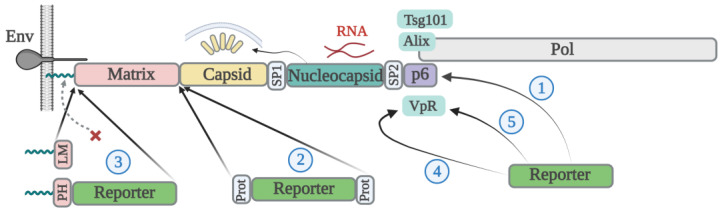
A simplified illustration schematically showing the function of each structural domain of HIV Gag (on top) and the approaches (1–5) used for reporter protein incorporation into viral particles (beneath). Each approach was described in the following references: 1—[25,26,27], 2—[29,30,31], 3—[34,35,36], 4—[39,41], and 5—[40], see main text. Myristate modification (shown by spirals) driven by signals from Lyn kinase (LM), or the phospholipase C-δ1 pleckstrin homology domain (PH) substitutes the matrix myristylation (shown by dashed arrow with X). Prot stands for HIV protease cleavage site. Prepared with https://app.biorender.com/illustrations/638a443e9cc25bb34b5c8f23 (accessed on 2 March 2023).

In conclusion, retroviral Gag is a self-assembling protein sufficient for particle formation. Cargo proteins can either be directly fused with a certain domain of Gag or indirectly incorporated into VLPs by binding to the Vpr or other viral non-structural proteins. However, Gag isolated from the viral genome needs to be codon optimized or trans-complemented by Rev and Rev response element (RRE) in order to be expressed efficiently. In addition, for VLP maturation, the presence of viral protease in trans or the insertion of a reporter gene into the *gag-pol* gene will be required. The approaches used to modify HIV Gag with cargo proteins are summarized in Figure 1.

## 3. Retroviral Disassembly and Cell Transduction Efficiency with Cargo Proteins

Although protein delivery via VLPs is a complex process that depends both on the payload in producer cells and release in target cells, we intentionally partitioned the discussion of the retroviral disassembly stage in a separate paragraph to understand the impact of a certain design on this mechanism. Followed by the fusion of mature HIV particles with the cellular membrane, MA mostly remains at the site of fusion while the capsid core is released into the cytoplasm, where it serves as a shell for the reverse transcription complex (RTC) and pre-integration complex (PIC), protecting them from innate immune sensing. Earlier, it was believed that reverse transcription is completed in the cytoplasm and that the core is too large to get through nuclear pores, and should be disassembled prior to PIC translocation into the nucleus. The latest data suggest that reverse transcription [45], as well as capsid core decay [46], are completed within minutes prior to viral dsDNA integration into the host genome. Thus, the intact HIV core mediates the nuclear translocation of the PIC, with the underlying mechanism remaining largely unknown. This is an important observation suggesting that all techniques facilitating the release of the Cas nuclease in the complex with the HIV capsid core (i.e., fused to Vpr or integrase (IN)) can be actively delivered to the nucleus.

It should be emphasized that the efficiency of protein release into the cytoplasm or nucleus largely depends on the mechanisms of cargo uptake/entry into the target cells and subsequent membrane fusion/penetration. Cationic lipids and CPP such as HIV trans-activator of transcription (Tat), Vp22, and Antennapedia peptide (Antp) initiate the uptake of transducing protein into endosomal vesicles. The subsequent protein release from the endosome to the cytosol or nucleosol can be problematic [47]. In contrast, VLPs utilize the specific and usually more efficient mechanism of membrane fusion mediated by viral Env and cellular receptor interaction. Thus, Kaczmarczyk et al. demonstrated that Cre recombination was much more efficient when Cre was fused to HIV Gag and delivered using VLPs pseudotyped with protein G from vesicular stomatitis virus (VSVG), than when Cre was fused to the protein transduction peptide from Tat [48].

The method used for packaging a transducing protein into VLPs also influences the kinetics of its release into the cytoplasm and trafficking. An interesting study has been performed in Gregory Melikyan’s lab. Two fluorescent proteins were packaged into VLPs, one being mCherry placed between MA and CA in the manner of the iGFP construct (see paragraph above), and the other being YFP fused to Vpr. Immediately after particle fusion, mCherry fluorescence was lost, whereas YFP puncta gradually became fainter over the first 20 min and then localized in the nucleus [49]. This implies that variants of Cas9 fused to Gag via a protease cleavage site will be rapidly released into the cytosol of target cells. In contrast, a Cas9-Vpr chimera fused directly will have delayed kinetics of release from the viral core; however, its nuclear import may be facilitated by Vpr, which has two nuclear localization signals (NLS) [50], as well as its association with PIC.

In contrast to the condensed viral core, immature HIV Gag-GFP, upon VLP fusion, stays attached to the cellular membrane and does not diffuse into the cytosol or get transported across the cytoplasm to the nucleus. This restricts the delivery of the cargo protein to only the submembrane space. Theoretically, a protein fused to Vpr can be released into the cytoplasm independently of the maturation status of VLPs, but without binding to a condensed core, its nuclear translocation can be reduced. It also should be noted that fusion with target membranes is inefficient using immature HIV particles constructed with HIV Env. Vpr acts early during virus entry and uncoating, and has an adverse effect on target cell proliferation by inducing cell cycle arrest and apoptosis [51,52]. This should be taken into consideration when delivering Cas RNP using the Vpr mechanism. On the other hand, Reuschl and coauthors recently showed that Vpr in the context of HIV reprograms naïve infected T cells to tissue-resident memory phenotype cells [53]. This phenotype is known to be more resistant to apoptotic signals, meaning that the survival of edited primary human lymphocytes may not be affected by Vpr.

**Figure 2:**
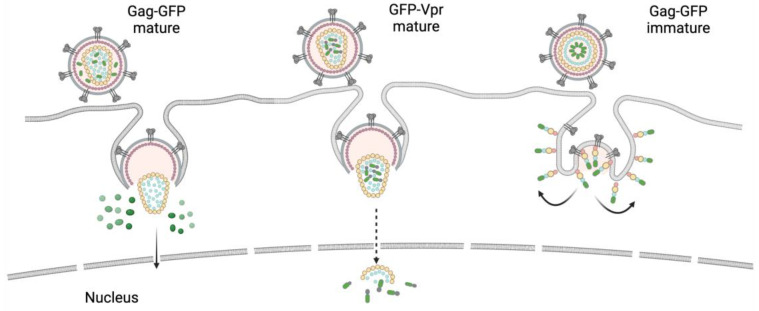
Three putative mechanisms of HIV VLPs fusion, disassembly, and reporter protein release in target cells depending on the VLP maturation status and cargo fusion with Gag or Vpr. The color codes of the structural domains of HIV Gag and reporter protein match those in the previous figure. Created with https://app.biorender.com/illustrations/638a443e9cc25bb34b5c8f23 (accessed on 2 March 2023).

In summary, the release of transducing protein from the carrier is an important aspect of its functioning inside the target cells. The mechanism of protein packaging dictates the mechanism of its uncoating. Most of the designs that rely on retroviral protease-mediated protein cut-off will result in the release of cargo protein inside the VLPs, which will then just be injected into the cytosol during the fusion process [54]. However, protease-mediated cleavage is often incomplete or causes cleavage of the cargo protein. Delivering proteins using Vpr avoids these problems as the release of cargo protein is based on core disassembly [38], the detailed mechanisms of which are not fully understood. A schematic showing the variants of reporter protein release at the virus entry stage is presented in Figure 2.

## 4. Biogenesis and Function of Exosomes

In addition to VLPs, different extracellular vesicles (EVs) had been widely used as a vehicle to deliver a variety of therapeutic molecules, including Cas9 RNPs. Unlike VLPs, they do not induce antiviral responses in cells. To better understand how the Cas9 RNP EV devised systems work, we first briefly describe the basic principles of EV formation and functioning.

All types of cells spontaneously produce EVs. This process is a part of complex intracellular sorting machinery that controls protein stability, renewal, and recycling. The major function of EVs is the elimination of unwanted molecules from cells. Besides being waste transporters, EVs mediate local and distant intercellular communication. EVs are classified into three main categories based on their size and mechanisms of formation: exosomes (30–150 nm), microvesicles (MVs) (100–500 nm), and apoptotic bodies (1–6 μm). Apoptotic bodies are formed from apoptotic cells, while MVs bud directly from the plasma membrane. Exosomes are the smallest EVs that are formed and released from cells as a result of inward budding into multivesicular bodies (MVBs) and their subsequent fusion with the outer membrane. There must be at least two different types of MVBs in a cell; one mediates fusion with lysosomes for the degradation of cargo accumulated within the intraluminal vesicles (ILVs) inside a cell, and the other bypasses this mechanism and transports ILVs to the plasma membrane for external secretion. Exosome biogenesis is a stepwise process that begins with the formation of molecular clusters consisting of lipids, transmembrane and cytosolic proteins, and RNA on the limiting membrane of MVBs. The key subunit hepatocyte growth factor-regulated tyrosine kinase substrate (Hrs) of the ESCRT-0 recognizes ubiquitinated proteins and sorts them into phosphatidylinositol-3-phosphate (PI3P)-enriched membrane domains (reviewed in [55]). Subsequently, ESCRT-0 interacts with tumor susceptibility gene 101 (Tsg101) and recruits ESCRT-I which, together with ESCRT-II, promotes inward membrane invagination around clusters of sorted proteins. ESCRT-III binds to ESCRT-II and recruits charged multivesicular body protein-4 (CHMP-4), which polymerizes around the neck of the budding ILV, following which CHMP-3 cleaves off ILV. Then, ESCRT-III disassembles with the aid of vacuolar protein sorting-associated protein 4 (Vps4). ALG-2 interacting protein X (Alix) is the adapter protein of ESCRT-III that facilitates ILV fission. Once loaded with ILVs, MVBs are transported to the plasma membrane along the actin and tubulin microfilaments using molecular motors and different types of Rab proteins. At the final step, MVBs fuse with the plasma membrane in a process mediated by soluble N-ethylmaleimide sensitive fusion protein attachment receptor (SNARE) proteins and release exosomes. The biogenesis of exosomes is a complex process that can also involve ESCRT-independent mechanisms, the sorting of non-ubiquitinated proteins, and the recruiting of tetraspanins and specific lipids (reviewed in [56]). These mechanisms determine the composition of exosomes, which are enriched in ESCRT proteins (such as Hrs, Tsg101, and Alix), different tetraspanins, α4 integrin, Lysosome-associated membrane glycoprotein 1 (LAMP-1), heat shock proteins, vinculin, flotillin, and arrestin. It should be noted that among the tetraspanins, only CD63 is specifically expressed on exosomes/lysosomes, and the others are widely present on the cell surface.

There is a certain interplay between the molecular mechanisms of HIV budding and exosome formation. The p6 domain of Gag contains Tsg101- and Alix-binding motifs that are both necessary for HIV virion abscission from the cell surface [57,58]. HIV-infected cells produce not only virions but also exosomes in large quantities. The composition of such exosomes differs from that of the exosomes produced by uninfected cells. The exosomes loaded with HIV negative regulatory factor (Nef) or Human T-lymphotrophic virus (HTLV-1) trans-activating protein Tax are capable of inducing pathological processes in non-permissive cells, which manifest in neurological disorders and malignancies (reviewed in [59]). Blood and all other biological fluids contain exosomes. Recipient cells uptake exosomes by endocytosis. How the content from exosomes then diffuses to the cytosol or nucleosol is not very clear. Sung et al. developed a pH-sensitive fluorophore pHluorin fused to CD63 to visualize the secretion of exosomes, which is accomplished by pH elevation, and their endocytosis by recipient cells that undergo gradual acidification [60]. If exosomes carry molecules that specifically interact with the cell surface receptors, they can trigger intracellular signal transduction and, for instance, immune cell activation. Alternatively, receptor-ligand interaction can mediate the fusion of exosomes with the plasma membrane, resulting in the release of soluble content into the cytoplasm and the lateral diffusion of exosomal transmembrane protein within the plasma membrane.

**Figure 3:**
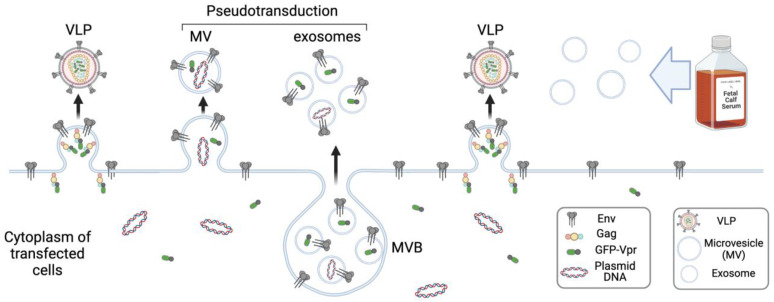
Contamination of VLPs with EVs and pseudotransduction. Transfected VLP-producing cells secrete MVs from the plasma membrane and exosomes as a result of MVB fusion with the plasma membrane. Once EVs are pseudotyped with Env, for example, VSVG, and spontaneously loaded with cargo protein, they can transduce (pseudotransduce) target cells along with VLPs. Created with https://app.biorender.com/illustrations/638a443e9cc25bb34b5c8f23 (accessed on 2 March 2023).

Thus, VLPs and exosomes share a certain level of similarity. They are difficult to separate from each other based on size or density gradient centrifugation, and immunolabeling them for surface-specific markers followed by magnetic bead isolation is considered the most appropriate method for their selection [61]. HIV hijacks endosomal sorting machinery for viral particle assembly [62], which in macrophages, can bud initially into MVBs and then release from cells upon MVB fusion with the plasma membrane [63]; exosomes do likewise. It is important to remember that HEK 293T cells that are widely used for VLP production will also produce exosomes which, together with the serum exosomes present in the culture medium, will contaminate viral preps obtained by centrifugation methods. Another feature of VLP generation is that producer cells shift secretion from MVs towards the smaller exosomes and actively export an excess of plasmid DNA with exosomes [64]. Collectively, these aspects of VLP preparation (Figure 3) for Cas RNP delivery should be taken cautiously as this will directly affect the mechanisms of gene editing.

## 5. Lessons from the Spontaneous and Specific Incorporation of Cas9 RNP into Exosomes

### 5.1. Nonspecific Loading of EVs with Cas9 RNP

In 2011, Mangeot and coauthors introduced the term gesicles to mean delivery vehicles that represent VSVG-pseudotyped extracellular vesicles, which are mainly exosomes that non-specifically incorporated a protein of interest. As exemplified by YFP and the receptor for ecotropic MLV mouse cationic amino acid transporter 1 (mCAT-1), both cytoplasmic and transmembrane proteins were efficiently packed and transduced target cells [65]. Interestingly, since VSVG mediates the fusion of exosomes with endosomal vesicles, the appearance of mCAT-1 on the surface of target cells indicates its active recycling from endosomes to the plasma membrane. In contrast, nucleus-localized tetracyclin transactivator (tTA) was poorly incorporated into gesicles, but its farnesylation [66] rescued this process. It is believed that non-vectorized spontaneous protein incorporation into gesicles is less effective than specific packaging. Nevertheless, these data explain earlier observations of bystander protein transduction (pseudotransduction) by unknown agents co-prepared with retroviral VLPs [67,68]. Later on, Montagna et al. demonstrated that gesicles are an efficient tool for Cas9 RNP delivery and gene editing when sgRNA is expressed in the cytoplasm under the control of the T7 promoter using a special packaging cell line, BSR-T7/5, with stable expression of T7 polymerase. The method was named VEsiCas [69]. Both studies confirmed that the expression of VSVG significantly increased cargo protein load into EVs, but the underlying mechanism remains unknown. Of note, the VSVG expression plasmid was used in these studies in much higher amounts than during classical lentiviral transduction, and so could be toxic for cells. In another study, a weak but still detectable editing activity transferred by EVs was observed in the coculture of cells transfected with the U6-sgRNA-Cas9 expression plasmid and recipient cells at a 4:1 ratio [70], a mechanism slightly resembling HIV cell-to-cell transmission, for the quantitation of which we have previously developed specific lentiviral reporter vectors [71,72]. Gene editing in this study was mediated by EVs without VSVG and abrogated in the presence of ceramide-dependent inward budding inhibitor GW4869. Similar EVs called GEDEX (genome editing with designed extracellular vesicles) produced by overexpressing only Cas9 and sgRNA in 293T cells had no advantage over gesicles, displaying modest gene editing results [73].

Thus, there are many obvious contradictions between these studies; for example, it is unclear whether the nuclear localization of Cas9 and U6-driven sgRNA interferes with their loading into EVs, and to what extent, how efficiently, and by what mechanisms the cargo is released in target cells without VSVG or any exogenous Envs. Nevertheless, these studies suggest that the level of passive, non-specific incorporation of reporter proteins and CRISPR/Cas9 into exosomes and microvesicles is quite significant, and should not be neglected when measuring vectorized specific protein loading into EVs or VLPs, which is discussed below. Moreover, the lack of linkage of Cas with carrier facilitates its rapid diffusion in target cells and its function at low concentrations.

### 5.2. Specific Mechanisms of Cas9 RNP Recruitment into EVs

One of the first studies showing a specific load of EVs with Cas9 RNP utilized the interaction between the PPXY domain of arrestin domain-containing protein 1 (ARRDC1) and the WW domain of the itchy E3 ubiquitin protein ligase (ITCH) protein of the neural precursor cell-expressed developmentally downregulated gene 4 (NEDD4) family. ARRDC1, like retroviral Gag, contains a C-terminal PSAP domain that recruits Tsg101 and the PPE(S)Y domains responsible for binding to WW domain-containing protein 2 (WWP2) and protein ubiquitination. Both motifs are required for virus budding and arrestin-mediated EV release that, unlike exosomes, occurs at the plasma membrane [74]. By fusing several WW domains with the N-terminus of Cas9 and co-expressing it with ARRDC1 and sgRNA, Wang et al. reported a two- to three-fold increase in WW-Cas9 EV load and GFP knockout (KO) efficiency in comparison to unmodified Cas9, suggesting a high level of spontaneous Cas9 packaging. Moreover, editing efficiency with EVs was about three-fold lower than that observed after direct plasmid transfection [75]. This can be explained by the lack of VSVG-mediated EV fusion and/or the specific mechanism for ARRDC1-Cas9 complex disassembly in target cells. The tetraspanin CD63, which is specifically expressed on late endosomes, exosomes, and lysosomes when C-terminally fused to GFP, can serve both as a tool for exosome visualization and distribution in vivo, and the exosome-mediated delivery of Cas9 C-terminally fused to an anti-GFP nanobody [76]. Consistent with the ARRDC1 study, Ye et al. observed a significant spontaneous load of CD63-GFP EVs with the wild-type (wt) Cas9 protein, but not with Cas9 mRNA, as well as a moderate increase in RNP incorporation and editing efficiency for Cas9-nanobody [77,78]. Perhaps, if the immune purification of EVs with anti-CD63 antibody was performed instead of physical separation, the observed effects would be more pronounced. The mechanisms of nanobody-GFP dissociation and Cas9 nuclear translocation in target cells were not investigated. However, the acidification of late endosomes may promote this process due to the pH sensitivity of the Ab-antigen interaction, which depends on histidines present in the active center of the antibody [79].

Alternatively, CD63-based recruitment of Cas9 into EVs can be achieved not only through protein-protein interaction, but also by using protein-RNA association. Yao and coauthors modified CD63 by appending the aptamer-binding protein Com to both the N- and C-termini of the protein and replacing stem loop two of the sgRNA with aptameric RNA com. Com-com interaction allows for the recruitment of Cas9 to EVs via sgRNA. This system demonstrated a two to five times more efficient recruitment of Cas9 from *Streptococcus pyogenes* (spCas9) and Cas9 from *Staphylococcus aureus* (saCas9), with com relative to spontaneous loading, and an up to 10 times higher gene editing efficiency by EVs added to target cells [80]. A substantial improvement, compared to previous systems, was achieved with two Com proteins fused with one molecule of CD63, and ensuring more efficient entry by pseudotyping EVs with VSVG. It remains unclear how U6-driven sgRNA, which stays in the nucleus, mediates CD63-Cas9 complex formation and why Pol-II-driven sgRNA expressed in the cytoplasm does not improve the system performance. Perhaps Cas9 molecules shuttle between the nucleus and cytoplasm, and the Cas9 molecules that capture sgRNA are recruited into EVs, whereas T7-driven cytoplasmic mRNA encoding sgRNA, which was described in Montagna’s work, may be weakly processed by ribozymes. A substantial drawback of all these EV systems for Cas9 RNP delivery is the lack of mechanisms providing editing complex release from a carrier molecule.

### 5.3. Reversible EV Systems for Packaging and Releasing Cas9 RNP

In this regard, molecular systems with regulated protein-protein interactions look more attractive. Campbell and coauthors engineered gesicles with chemically inducible recruitment of the Cas9 RNP complex. They fused the C-terminus of the CherryPicker Red transmembrane fluorescent protein with 12-kDa FK506-binding protein (FKBP12) and linked Cas9 with the FKBP-rapamycin-binding (FRB) domain of the mammalian target of rapamycin (mTOR) kinase. CherryPicker Red is present on EVs. Once rapamycin or its paralogs, such as AP21967, is added to the culture of producer cells, FRB heterodimerizes with FKBP12, and Cas9 is recruited into CherryPicker-positive gesicles. Upon the treatment of target cells with gesicles, the content of EVs is released into the cytoplasm, the heterodimerizer is diluted, and Cas9 dissociates from CherryPicker. Despite being a smart concept, this design demonstrated only a two-fold enrichment of gesicles with Cas9 protein level, and a moderate enhancement in HIV proviral DNA editing efficiency [81]. It is unclear why gesicles in this study were not isolated using anti-Cherry Red antibody that theoretically should concentrate its active fraction loaded with Cas9 RNP. Recently, Osteikoetxea et al. compared several light or chemically inducible heterodimerizing systems in combination with different exosome-specific molecules, and found that Cas9 fused to cryptochrome 2 (CRY2) paired to the palmitoylated/double myristoylated variant of cryptochrome-interacting basic helix-loop-helix 1 (CIBN), or fused to CD9 most effectively loaded Cas9 to EVs and edited target genes [82]. The FKBP-FRB chemical dimerization system performed less efficiently with different EV-specific proteins relative to the CIBN-CRY2 photosystem. Again, the authors did not show how significantly dimerization increased the Cas9 payload, only mentioning a high level of spontaneous Cas9 incorporation in the discussion, and did not use VSVG or the expression of sgRNA in the cytoplasm.

### 5.4. Summary

The non-specific spontaneous incorporation of Cas9 RNP or any other cargo proteins is quite high, as confirmed in many reports, and should be taken into consideration during the development of specific packaging systems. The addition of VSVG expression plasmid to the transfection formula of EV-producing cells greatly improves overall system performance through at least three distinct mechanisms: increased EV production, efficient EV fusion with target membranes, and, related to this process, EV escape from lysosomal degradation in target cells [83,84,85]. In comparison to VLPs, the level of EV production is lower, so methods boosting this process are very desirable, such as the expression of a six-transmembrane epithelial antigen of prostate 3 (STEAP3), syndecan-4 (SDC4), and fragment of L-aspartate oxidase (NadB) from the tri-cistronic construct that has been reported to enhance EV production from 15- to 40-fold, and warrant EV usage without concentration [86]. It appears that recruiting Cas9 into EVs via sgRNA is a more efficient approach than protein-protein interaction, which can be explained by the selective packaging of Cas9 RNP and not just Cas9 protein, although the mechanisms of sgRNA delivery from the nucleus to the site of assembly remain unclear. In this context, making the protein-RNA interaction inducible, as was shown for the tetracycline-dependent repressor (TetR) aptamer [87], will additionally improve the system by enabling efficient cargo release in the cytoplasm. There are many efficient methods for protein sorting into EVs, such as fatty acid modification, palmitoylation, and myristoylation at the N-terminus or farnesylation at the C-terminus of the protein. Additionally, cargo proteins can be linked to the cytoplasmic tails of the tetraspanins CD63 and CD9 for efficient recruitment to exosomes. Finally, the immune purification of EVs and the use of an exosome-free medium are helpful to get rid of ballast non-active EVs that can interfere with protein transduction. The pros and cons of the current methods used for EV load with Cas9 RNP are summarized in Table 1.

## 6. Retroviruses for the Delivery of Cas9 RNP: Restrictions and Evolution of Design

### 6.1. Fusion of Cas9 with Gag for Delivery with VLPs

Unlike EVs, retroviruses are tightly packed with core and other viral proteins, which creates significant challenges for the incorporation of external proteins, especially a large spCas9 nuclease. Early work by Choi et al. demonstrated that linking Cas9 to the N-terminus of HIV Gag-Pol through a protease cleavage site and pH myristoylation signal preserves Gag assembly, although at a two-to-five-fold lower level of efficiency compared to standard lentiviral (LV) delivery. Complementing this construct with VSVG, wt HIV packaging vector, and transfer plasmid encoding U6-sgRNA generated LV particles capable of Cas9 protein transduction and the integration/stable expression of sgRNA in target cells. The particles displayed on-target editing activity (C-C chemokine receptor 5 (CCR5) and HIV provirus KO) at a slightly lower level than that which gives stable CRISPR/Cas9 expression, but no detectable activity at off-target sites [88]. A more popular method for Cas9 encapsidation is linking it to the C-terminus of Gag and separating by protease cleavage site combined with the transfection of a helper packaging plasmid, which supports VLP production and maturation. Thus, Montagna et al., in the work discussed above [69], created the HIV-1 Gag-Cas9 and MiniGag-Cas9 [89] fusion constructs to deliver Cas9 with VLPs, and compared them with pseudotransduction mediated by VEsiCas, which are usually co-purified with VLPs. They introduced a cryptic start codon between the protease cleavage site and Cas9 so that more than half of the amount of Cas9 protein expressed by the transfected producer cells was not linked to Gag or MiniGag, allowing it to be passively packaged into gesicles instead of VLPs. The results demonstrated no differences in editing activity between samples with or without the cryptic start codon, suggesting that the low level of Gag-Cas9 caused by the ATG codon addition is compensated by a high level of Cas9 delivered with gesicles. This is a very important notion underlying the significant role of EVs in the generation of VSVG-pseudotyped Cas9-VLPs. Impressive results were reported on so-called Nanoblades consisting of MLV Gag fused to Cas9 via the MLV protease cleavage site [90]. In most cases, the levels of gene editing or activation with Nanoblades in primary human cells, mouse cells, and zygotes exceeded those detected after Cas9 RNP electroporation. To achieve VLP transduction of resting primary cells that do not express a VSVG receptor LDL (low-density lipoprotein) [91], the authors elaborated on co-pseudotyping VLPs with the baboon retroviral envelope glycoprotein (BaEV) [92]. They also reported the transfer of about 6.5% of luciferase protein activity from 293T producer cells to target cells, as well as a weak transfer of the tetraspanin CD81, which can be passively incorporated into Nanoblades and/or co-purified EVs. Despite the impressive results, we were not able to adapt this technology for editing important HIV genes (unpublished data). From this work, it remains unclear how the spatially separated Gag-Cas9, which is myristoylated and anchored to the plasma membrane, and the Pol III-transcribed sgRNA localized in the nucleus interacted with each other, and how specific and efficient the cleavage of Gag-Cas9 by MLV protease was.

### 6.2. Advanced and Combined Packaging Systems: Dimerization Domains, Vpr, RNA Transduction

A more complex and advanced delivery system based on FKBP12-FRB heterodimerization and RNA recruitment named NanoMEDIC has been engineered by Gee and coauthors [93]. In the first step, they tested the packaging of FRB-Cas9 into EVs by attaching FKBP12 to the C-terminus of the VSVG or LM myristoylation signal as well as into VLPs by embedding FKBP12 between the LM and HIV Gag. Compared to EV approaches, HIV VLPs displayed the highest level of Cas9 packaging and editing activity, which can also result from more robust particle production by viral Gag over EV cellular machinery. To further enhance Cas9 RNP encapsidation, U6-sgRNA was substituted with Tat-dependent construct LTR-Ψ-HH-sgRNA-HDV that is expressed in the cytoplasm from long terminal repeat (LTR) promoter, packaged through the HIV Ψ-signal, and processed in parallel by hammer head (HH) and hepatitis D virus (HDV) ribozymes releasing active sgRNA. Interestingly, the RNA packaging mechanism appeared to be more significant for the editing activity of NanoMEDIC than AP21967-induced protein-protein interaction. Thus, heterodimerization increased GFP editing three-fold, whereas the co-expression of LTR-Ψ-HH-sgRNA-HDV in producer cells and U6-sgRNA in target cells improved gene editing more than 10-fold. This indicates that many molecules of Cas9 recruited into VLPs by protein interaction are likely devoid of sgRNA, and any mechanisms enhancing sgRNA packaging in producer cells or its expression in target cells will increase Cas9 RNP formation and gene editing. The deficiency of sgRNA molecules in the cytoplasm of producer cells can be caused by reduced ribozyme-mediated RNA processing, especially for HDV [94], and by the nuclear localization of Pol III-driven sgRNA [95]. It is worth noting that NanoMEDIC are immature HIV VLPs and benefit from the absence of viral protease that can non-specifically cleave Cas9. However, immature HIV Gag does not allow the HIV envelope protein to function properly, making cell-specific delivery with NanoMEDIC problematic. In contrast, Hamilton et al., using Cas9 fused with the C-terminus of HIV Gag separated by the HIV protease cleavage site in combination with a helper packaging vector, lentiviral transfer vector, and Env expression plasmid, generated infectious VLPs capable of the simultaneous transfer of Cas9 RNP and stable integration of the lentiviral vector. This technique was perfectly matched to chimeric antigen receptor (CAR) T-cell preparation as it enabled the integration of the CAR expression cassette and endogenous T cell receptor (TCR) or major histocompatibility complex I (MHC-I) KO at the same time. By pseudotyping VLPs with HIV Env, they demonstrated the possibility of β-2 microglobulin KO specifically in CD4 lymphocytes in a mixture with CD8 lymphocytes [96]. Consistent with the above-mentioned phenomenon of the incomplete loading of Cas9 with sgRNA during VLP production, the authors noted a decreased editing efficiency for VLPs produced with the conventional U6-sgRNA plasmid relative to VLPs generated with the lentiviral U6-sgRNA vector, which elicited sgRNA expression both in producer and target cells. Indikova and a colleague engineered HIV VLPs in which Cas9 incorporation was mediated by fusion with the C-terminus of Vpr [97]. The encapsidation was enhanced by adding the HIV RRE/Rev mechanism of RNA nuclear export and its co-localization with HIV Gag [98] but not with the constitutive transport element (CTE) export signal from Mason-Pfizer monkey virus (MPMV). The system worked with the lentiviral U6-sgRNA cassette and the intergrase-deficient or -proficient packaging vector, but the reverse transcriptase (RT) inhibitor azidothymidine completely blocked its editing capacity, suggesting that VLPs pack only Cas9 protein, and sgRNA is expressed exclusively in transduced cells. From this study, the degree of specific Cas9 recruitment by Vpr is unclear as no comparison was made to the mutants Vpr E25K or H33L that lack the interaction with p6 [99].

### 6.3. Packaging of Cas9 RNP via sgRNA

An alternative method for Cas9 RNP encapsidation through aptamer RNA-protein binding has been explored in two sequential studies by Baisong Lu and coauthors, who also applied this method for EV loading (discussed above). First, they tested different HIV viral proteins for MS2 binding protein (MS2-BP) incorporation. Specifically, MA with 44–132 a.c. substitution and NC with insertion downstream of the second zinc finger were made in the context of a full packaging vector, while mutant Nef (G3C V153L G177E) with increased incorporation and Vpr were C- and N-terminally fused to MS2-BP, respectively, and co-expressed in separate expression vectors. MA modification reduced VLP production. Consistent with the numbers of protein molecules packaged into VLPs, their gene editing activity was in the order Vpr < Nef < NC [100]. In a subsequent study that compared replacing the tetraloop with the aptamer MS2, inserting the aptamer into Stem Loop II, or appending the aptamer to the 3′-end of sgRNA, the replacement of the tetraloop with aptamer was shown to preserve sgRNA function. Among the four tested aptamers, MS2, PP7, BoxB, and com, which were placed in sgRNA instead of the tetraloop, com demonstrated the highest activity of sgRNA. Similar to data obtained on EVs, the combination of NC-Com with sgRNA-com(+) increased the GFP editing rate of HIV VLPs about 10-fold over the editing efficiency of control sgRNA-com(−) [101], outperforming other vectorized Cas9 RNP systems. It remains unclear how significantly the com aptamer influences the level of Cas9 protein in VLPs as the authors provide conflicting results, but at least changes in protein levels are not as impressive as those in the editing data. Once again, this suggests that the Com-com interaction mechanism under conditions of U6-sgRNA deficiency in the cytoplasm is important for recruiting functional Cas9 RNP rather than the total level of the Cas9 protein. Except for NC-com cleavage in released VLPs, the mechanism of system disassembly in target cells remains speculative, but may involve the nuclear translocation of CRISPR/Cas9 within the capsid core.

### 6.4. Summary

Induced by retroviral Gag, VLP assembly is a significantly more powerful process in comparison to EV biogenesis. However, it should be remembered that the physical separation of VLPs and exosomes is impossible, so the cumulative editing potency of isolated nanoparticles will be determined by EVs as well if they are co-loaded with Cas9 RNP. The fusion of the Cas9 protein with Gag directs nuclease movement exclusively to VLPs. All the other approaches provide a window for Cas9 to be packaged into EVs. Due to its large size, the fusion of Cas9 with Gag often reduces the level of VLP production, which can be rescued by a helper vector; however, wt Gag complementation leads to a decrease in VLP payload. In this regard, protein modules like Com, FRB, and Vpr benefit from their small size, but the efficiency and specificity of Cas9 incorporation will depend on the ratio between assembling elements and their spatiotemporal characteristics, which are discussed in the next paragraph. Thus, if we assume that the sgRNA is deficient in the cytoplasm due to nuclear localization (U6-sgRNA), poor processing from precursor RNA, or fast degradation, then the recruitment of Cas9 through aptamer-modified sgRNA will be the best method to obtain functionally active VLPs; this has been demonstrated in many reports. Protein-protein interaction methods require an excess of cytoplasmic sgRNA for Cas9 RNP incorporation, the achievement of which has been attempted during NanoMEDIC development. An equally important aspect of this technology is the mechanism of VLP disassembly. For Gag-Cas9 fusions, the release of Cas9 depends on viral protease activity that can be insufficient at the target site and excessive at off-target sites, but can be improved overall, as demonstrated recently for eVLPs [102]. Chemical- or photo-inducible protein heterodimerizing modules are efficient and have a clear mechanism of action. It should only be mentioned that very active substances like rapamycin and its paralogs require complete removal from VLPs by repeated washes before transduction. The small HIV non-structural proteins, Vpr, Nef, and integrase, that are naturally packed into virions, will likely retain Cas9 RNP in a complex with a capsid core until transportation to the nucleus, unless an additional dissociation mechanism to release Cas9 is provided. The current methods for VLP-based delivery of Cas9 RNP, as well as their advantages and disadvantages, are shown in Table 2.

## 7. From Particle Assembly to Gene Editing: Important Considerations for Making the System Work Perfectly

### 7.1. Non-Specific Incorporation

We intentionally reviewed the current literature on Cas9 RNP VLPs together with EVs, and the first question that should be addressed when designing a delivery system is where the Cas9 protein and sgRNA go. The most popular packaging cell line, HEK 293T, produces high levels of proteins in the cytoplasm and releases MVs and exosomes. In this regard, except for the direct fusion of Gag or an EV-specific molecule with Cas9, all other designs are prone to sending Cas9 to VLPs, MVs, or exosomes, and this should be investigated by the separation of VLPs from EVs or MVs from exosomes. Why is this possible? The explanation is given below.

### 7.2. Stoichiometry

The equimolar concentration of two molecules maximizes the formation of conjugates between them. The component in cells that is in excess will exist predominantly in a conjugate-free form that helps to bypass the specific recruiting mechanism and increase its spontaneous loading into extracellular nanoparticles. At least three components, carrier protein, Cas9, and sgRNA, participate in Cas9 RNP particle formation, for which an equal molar ratio in cells is difficult to attain. Therefore, if the level of sgRNA is low in the cytoplasm, then the recruiting of Cas9 via a sgRNA-aptamer, such as com, would be advantageous over the protein-protein mechanism of interaction, since more RNP will be packaged. The other way around, protein dimerization should work perfectly when there is plenty of sgRNA in the cytoplasm, as sgRNA or mRNA do not tend to be packed spontaneously into EVs or VLPs [77,78]. While the optimal (equimolar) ratio of these components is often achieved empirically by titrating plasmids, their accurate measurement in the cytoplasm would help understand the real situation.

### 7.3. Temporal Characteristics

Cas9 and its fusion products are large proteins so their expression will be delayed relative to smaller proteins or sgRNA. It was noted that the transfection of the VSVG vector into 293T cells stably expressing GFP yielded more fluorescent gesicles than the co-transfection of two coding plasmids in 293T cells [65]. This can be explained by the production of empty VSVG-gesicles early after transfection, which later during entry may interfere with gesicles loaded with cargo. The practice of changing the medium and harvesting VLPs/EVs at a later time point may help circumvent this shortcoming.

### 7.4. Spatial Issue (sgRNA)

All interacting elements should be present at the site of particle assembly, i.e., in the cytoplasm, in close proximity to the outer or endosomal membrane. As emphasized earlier, widely used U6-controlled sgRNA is expressed in the nucleus and likely appears in the cytoplasm at a reduced level due to the shuttling of Cas9 between the nucleus and cytoplasm, which makes for a functional system but is far from being ideal. The problem can be solved by cytoplasmic expression from the T7 [69] or Pol II promoter with the subsequent cutting out of sgRNA from a long mRNA transcript, which, however, can pose new problems. The currently tested approaches for sgRNA processing such as ribozymes [93], transport RNA (tRNA) [103], and potentially short hairpin RNA (shRNA) [104] either are not so highly effective, or the synthesized sgRNA is unstable when not bound to the Cas9 protein [95]. In this regard, Cas12a (Cpf1), which is able to process the crRNA cassette at a high level of efficiency and is perfect for the multiplexing of gene editing [105,106], can also protect processed cytoplasmic sgRNA from degradation by immediate interaction with it.

### 7.5. Spatial Problems (Cas9)

Cas9 and other dsDNA-binding nucleases must go to the nucleus in target cells to bind and edit genomic DNA, but should localize in the cytoplasm of particle-producing cells. Directly fused Gag and Cas9 counteract each other for the resulting subcellular localization as Gag is anchored to the plasma membrane through its myristate moiety, whereas NLS forces Cas9 to move to the nucleus. Recently, Banskota et al. applied a smart design to shift MLV Gag-Cas9 localization toward the cytoplasm in producer cells. The previously described Nanoblades were transformed to more efficient eVLPs by inserting a nuclear export signal (NES) downstream of Gag and upstream of the optimized protease cleavage linker and NLS-BE-Cas9-NLS, which increased the cytoplasmic localization of fusion protein and its load into VLPs 1.3- and 10-fold, respectively. Followed by VLP budding, MLV protease cleaved the polyprotein into Gag-NES and NLS-BE-Cas9-NLS, allowing BE-Cas9 to be efficiently released and enter the nucleus in transduced cells, which resulted in the improvement of eVLP base editing efficiency by eight-fold compared to Nanoblades [102]. As an alternative to the protease-mediated regulation of Cas9 localization, the photo-inducible NLS module LINuS [107] or NES module LEXY [108], or their analogs, called LANS and LINX [109], respectively, can be added to Cas9 to increase its cytoplasmic localization in producers and nuclear traffic in target cells. A slightly different mechanism of nuclear-cytoplasmic regulation was implied in the chemically induced iCas9 system, where Cas9 was flanked by ERT2 (mutant version of the receptor-binding domain of the estrogen receptor), which binds to heat shock proteins and retains Cas9 in the cytoplasm. Upon the addition of 4-hydroxytamoxifen, this interaction is abolished and Cas9 is translocated into the nucleus [110]. These regulatory modules can be adapted for improving Cas-VLP/EV assembly/disassembly rates. Despite their large size, especially ERT2, relative to the protease cleavage site, they provide more robust and tunable regulation of Cas9 localization.

### 7.6. Size of the Cas Proteins

The larger the protein, the greater the challenge for efficient packaging. The widely used spCas9 is the 1368-amino-acid nuclease, which exceeds the size of HIV Gag by almost three-fold. Moreover, its fusion modifications such as base editors or prime editors are even larger. To improve payload, it is logical to use the smallest nucleases, but the problem is that most are poorly adapted for eukaryotic and mammalian genome editing. As already discussed, the Cpf1 nuclease has the advantage that it can be expressed with crRNA from a single vector and mediate crRNA processing, but it is only slightly shorter than spCas9. Among the smallest Cas proteins, some have been recently re-engineered to work well in human cells, including the Cas9 ortholog Cje3Cas9 from *Campylobacter jejuni*, <1000 aa [111], the type V nuclease CasΦ-2 (Cas12j2) isolated from a huge bacteriophage (757 aa [4,112]), and Un1Cas12f from an uncultured archaeon (529 aa [113,114,115]). These miniature Cas nucleases can be chosen as candidates for future Cas-VLP/EV engineering.

### 7.7. Recruiting Mechanism

Retroviral Gag is a good target for Cas protein encapsidation as it efficiently self-multimerizes and buds from cells. Recently, Segel et al. identified the Gag homolog of paternally expressed gene (PEG10) from the mouse and human LTR retrotransposon involved in mammalian placenta formation as a suitable tool for VLP formation and the delivery of therapeutic genes [116]. The endogenous nature of PEG10 warrants low immunogenicity compared to viral Gag, which will facilitate the adoption of PEG10 for Cas RNP delivery. In this regard, targeting gesicles/exosomes/microvesicles, for example, via CD63 or fatty acid modification of proteins, has the same advantages over retroviral VLPs, but requires EV boosting methods to achieve a high level of production [86]. As discussed above, the mechanism of RNP recruitment through protein or RNA depends on carrier-Cas-sgRNA stoichiometry, but also should be selected based on the capacity for system disassembly in target cells (see discussion below).

### 7.8. Entry

The fusion of nanoparticles with the target membrane determines the efficacy and specificity of Cas9 RNP delivery. Currently, VSVG outperforms the other viral Envs in efficiency but does not provide specificity. The benefit of VLPs/EVs compared with many other methods of delivery is that they can be easily pseudotyped with a certain Env and, therefore, utilized for cell- and tissue-specific in vivo gene editing. However, there is a nuance. While HIV Gag and Env exploit a natural mechanism of HIV VLP entry that has been shown to mediate efficient and specific gene editing of CD4 lymphocytes with Cas9-VLPs [96], the usage of EVs or pseudotyping VLPs with heterologous Env eliminates this natural mechanism. This does not mean that these Envs will not mediate fusion at all, but the improvement of this mechanism through Env reconstruction with a heterologous intracellular domain might be necessary. Other characteristics of Env such as immunogenicity and the ability to mediate particle fusion with primary cells are of importance. Thus, finding that the endogenous Env protein syncytin A (SYNA) can support CRISPR/Cas9 mRNA delivery with PEG10 VLPs as efficiently as VSVG [116] can advance the in vivo application of SYNA-PEG10 by greatly reducing particle immunogenicity, whereas BaEV Env is likely to be helpful for the ex vivo therapeutic gene editing of primary cells that widely express the receptor for this Env [90,92].

### 7.9. Disassembly

**Figure 4:**
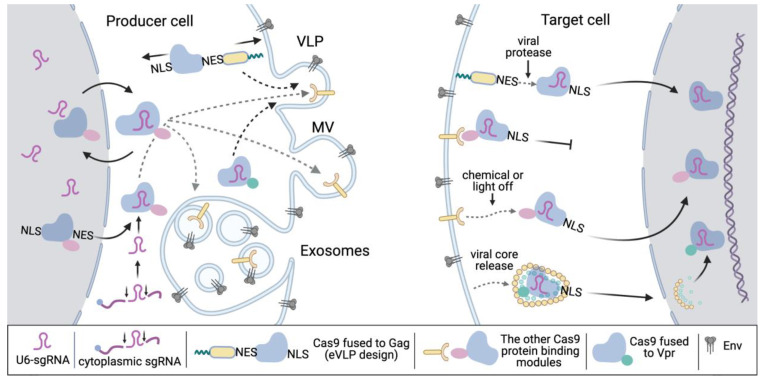
The mechanisms of Cas9 RNP recruitment into VLPs and EVs (on the left) and release in target cells (on the right) used by different delivery systems. The arrows indicate intracellular movements of the molecular complexes. The potential problems related to these mechanisms are schematically shown as well and include the nuclear localization of sgRNA and Cas9 in producer cells, the nonspecific incorporation of Cas9 into MV, exosomes, or VLPs, and opposing localization signals in hybrid molecules. In target cells, there can be no specific mechanism of Cas9 RNP release into the cytoplasm and trafficking to the nucleus or unclear mechanism of Ca9-Vpr viral core disassembly. The image is created with The image is created with https://app.biorender.com/illustrations/638a443e9cc25bb34b5c8f23 (accessed on 2 March 2023).

Unlike EVs, VLPs bear a capsid core that is normally released after virion fusion, shields the RTC and PIC, and transports complexes to the nucleus. Theoretically, this mechanism can help move core-packed CRISPR/Cas9 to the nucleus and facilitate genome editing even though Cas9 lacks NLS. In practice, no one has investigated this. The extent to which the core functions during transduction by VLPs depends on VLP design. Any EVs and immature VLPs lack this mechanism, and Cas9 RNP should be released into the cytoplasm as a result of viral protease cleavage, dilution of the heterodimerizing reagent, or altered complex photostability. In the case of using mature VLPs, especially those with the *pol* gene and that are fully infectious, the core may play a certain role in Cas9 RNP trafficking from the site of VLP fusion to the nucleus. This is possible for Com-com interaction methods [101] and the fusion of Cas9 with Vpr [97] since, following Gag processing, the resulting complex NC-Com-sgRNA-com-Cas9 will interact through NC with CA, and Vpr-Cas9 through p6 will stay in the core. Once transported to the nucleus, the core decays rapidly; however, RT and pre-integration events should precede this [46]. Thus, it is not clear whether the core in the nucleus will be able to release passenger CRISPR/Cas, and whether Vpr or NC appended to the Cas RNP will interfere with the gene editing activity of the complex after release. The addition of a protease cleavage site between NC-Com and Cas9-Vpr and a comparison of the editing activity of RNPs with and without this modification will shed light on this mechanism. Indeed, Indikova and a colleague included an HIV protease cleavage linker between Vpr and Cas9, but whether it elevates or decreases the editing activity of Cas9-VLPs was not studied [97]. In contrast, EVs cannot be designed using a protease cleavage site. How Cas9 RNP works in target cells without a specific release mechanism remains unclear, but the inclusion of mechanisms such as chemical- or photo-inducible heterodimerization is anticipated to enhance system efficacy. The mechanisms and potential problems related to the recruitment and release of Cas9 RNP during VLP/EVs engineering are summarized in Figure 4.

## 8. Conclusions

By critically analyzing the current and past literature on the mechanisms of extracellular particle assembly and disassembly for the delivery of the Cas RNP complex, we drew the reader’s attention to the most problematic aspects of this process from the step of producer cell transfection to RNP nuclear entry in target cells. First, we envisioned that exosomes would have a clear advantage over VLPs for therapeutic application due to their much lower immunogenicity, but the titer of EVs should be substantially boosted to be comparable with the level of VLP production, and this can be achieved by co-expressing STEAP3, SDC4, and NadB. Since the high and steady-state expression of sgRNA in the cytoplasm remains challenging, the most effective mechanism of Cas9 RNP loading onto exosomes is the binding of sgRNA to CD63 tetraspanin via aptamer Com. This method can be further improved by substituting aptameric RNA com with TetR, whose interaction with the respective TetR protein is regulated by tetracycline, which will allow CD63-sgRNA/Cas9 complex assembly in producer cells and disassembly in transduced cells. Second, a good and safe alternative to exosomes could be PEG10 VLPs pseudotyped with SYNA envelope protein. The human origin of SYNA-PEG10 will ensure both the lowest immunogenicity and high level of viral particle production. Third, the problem with the packaging of a large Cas9 protein could be solved by the usage of recently discovered miniature type V Cas nucleases and their adaptation to human genome editing. Finding mini-Cas proteins with CRISPR RNA processing ability like Cas12a will also help circumvent the limitations related to low cytoplasmic sgRNA processing/instability. Once a high level of sgRNA in the cytoplasm is achieved, a method of protein-protein interaction mediating complex assembly and recruitment into particles and subsequent release into target cells, such as NanoMedic or viral protease-based molecular cleavage, can be chosen. Fourth, considering that VLP production in 293T cells is accompanied by the release of EVs, the combined technology providing Cas9 RNP loading in both VLPs and EVs may advance the editing potency of bulk particles harvested from producer cells.

## Figures and Tables

**Table 1:** Current systems for delivery of Cas9 RNP using EVs. The pros and cons of each system are indicated by underlining or italicizing, respectively.

Name	Mechanism of Assembly	Mechanism of Disassembly	Features	Ref.
1. VEsiCas	*Absent*	Spontaneous release	*No VSVG*, sgRNA under T7 promoter	[69]
2. GEDEX	*Absent*	Spontaneous release	*No VSVG, U6-sgRNA*	[73]
3. ARMMs	ARRDC1 + WW-Cas9	*Absent*	*No VSVG, U6-sgRNA*	[75]
4. CD63	CD63-GFP + Cas9-anti-GFP-nanobody	*Absent*. Acidification in late endosomes?	*No VSVG, U6-sgRNA, no CD63^+^ exosome isolation*	[77,78]
5. Aptamer com	Com-CD63-Com + sgRNA-com	*Absent*	VSVG, *U6-sgRNA*, Cas9 recruitment via sgRNA	[80]
6. Cherry Picker Red	CherryPicker-FRBP12 + Cas9-FRB, chemical	Removal of chemical dimerizer	VSVG, *U6-sgRNA, no CherryPicker^+^ exosome isolation*	[81]
7. CIBN-CRY2	Palm(Myr)-CIBN (CD9-CIBN) + Cas9-CRY2, light	Turning off the light	*No VSVG, U6-sgRNA*, comparative study	[82]

**Table 2:** Current systems for delivery of Cas9 RNP using VLPs. The pros and cons of each system are indicated by underlining or italicizing, respectively.

Name	Mechanism of Assembly	Mechanism of Disassembly	Features	Ref.
1. PH-Cas9-GagPol	Fusion to the N-terminus of HIV GagPol	Cleavage by HIV protease	Lentiviral U6-sgRNA delivery to target cells	[88]
2. Gag-Cas9, MiniGag-Cas9	Fusion to the C-terminus of HIV Gag		*Co-delivery of Cas9 to VLPs and EVs* *, U6-sgRNA*	[89]
3. Nanoblades	MLV Gag-Cas9 fusion	Cleavage by MLV protease, *low level*	BaEV Env, *U6-sgRNA*	[90]
4. NanoMEDIC	LM-FKBP12-HIV-Gag + FRB-Cas9 (chemical) and LTR-Ψ-HH-sgRNA-*HDV* (RNA)	Removal of chemical dimerizer AP21967	Comparative study, double recruiting mechanism, *not with HIV Env*	[93]
5. HIV Gag-Cas9	Fusion to the C-terminus of HIV Gag	Cleavage by HIV protease	*U6-sgRNA*, CAR transduction, delivery to CD4 lymphocytes	[96]
6. Vpr-Prot-Cas9	p6-Vpr interaction and protease cleavage	Cleavage by HIV protease + core release	*Parallel lentiviral transduction,* *no packaging of sgRNA*	[97]
7. Aptamer *com*	NC-Com + sgRNA-*com* (RNA)		*U6-sgRNA*, Cas9 recruitment via sgRNA	[101]
8. eVLP	MLV Gag-Cas9 fusion + NES	Cleavage by MLV protease, optimized	*U6-sgRNA*, cleavable NES	[102]
